# Single‐Cell Analysis of Hepatic Non‐Parenchymal Cells Reveals Endothelial Dysfunction and Altered Endothelial and B Cell Communication in Acute‐on‐Chronic Liver Failure

**DOI:** 10.1155/jimr/5421823

**Published:** 2026-08-31

**Authors:** Jie Jia, Yuehong Dong, Yu Zhao, Shaoyou Li, Tangwei Mou

**Affiliations:** ^1^ Research Center for Clinical Medicine, First Affiliated Hospital of Kunming Medical University, Kunming 650032, China, kmmc.cn

**Keywords:** acute-on-chronic liver failure, endothelial dysfunction, immune microenvironment, intercellular communication, single-cell RNA sequencing

## Abstract

Acute‐on‐chronic liver failure (ACLF) is a fatal syndrome defined by hepatic decompensation and systemic inflammation, yet the underlying cellular networks remain elusive. Here, we established an ACLF mouse model recapitulating clinical hallmarks and performed single‐cell RNA sequencing (scRNA‐seq) on hepatic non‐parenchymal cells (NPCs). We identified 30 cell clusters and observed profound spatial remodeling of NPCs driven by pro‐inflammatory mediators. Endothelial cells (ECs) underwent a critical transition from homeostasis to dysfunction, marked by mitochondrial damage and activation of NF‐κB and MAPK pathways. Ligand–receptor interactome analysis identified dysfunctional ECs as central hubs driving global network reconfiguration, primarily via the *Lgals9–Ighm/Cd45/Cd44* axes, alongside the suppression of homeostatic signals (*Cd55* and *APP*). Furthermore, we mapped B cell lineage trajectories and validated the spatial co‐localization of EC and B‐cell interactions through Lgals9–Ighm. This study defines the EC and B‐cell communication landscape and suggests that the Lgals9–Ighm axis may play a critical role in ACLF progression, representing a potential candidate for therapeutic intervention.

## 1. Introduction

Acute‐on‐chronic liver failure (ACLF) is a distinct clinical syndrome characterized by acute hepatic decompensation in patients with pre‐existing chronic liver disease, accompanied by rapid development of organ failures and high short‐term mortality [1]. Large clinical studies and international guidelines recognize ACLF as a dynamic condition driven by precipitating events superimposed on chronic liver injury, with limited effective therapeutic options beyond supportive care and liver transplantation [1–4]. Despite advances in intensive care, the prognosis remains poor, highlighting the urgent need to elucidate the cellular and molecular mechanisms underlying ACLF pathogenesis.

Accumulating evidence indicates that ACLF represents a complex immunopathological state characterized by profound systemic inflammation and immune dysregulation [1, 3–5]. Recent mechanistic analyses further suggest that immune activation and impaired immune regulation coexist during disease progression, contributing to organ failure [5]. Single‐cell multimodal profiling of hepatitis B virus (HBV)‐associated ACLF has revealed dynamic remodeling of multiple immune compartments during disease progression [6], while peripheral blood single‐cell transcriptomic studies have demonstrated marked T‐cell exhaustion in patients with HBV‐associated ACLF [7], underscoring the central role of immune dysfunction in ACLF.

The liver harbors a highly specialized immune microenvironment composed of hepatocytes and diverse non‐parenchymal cells (NPCs), including endothelial cells (ECs), hepatic stellate cells, macrophages, and lymphocyte subsets [8]. Coordinated interactions among these cellular populations are essential for maintaining hepatic homeostasis, whereas disruption of these networks promotes inflammation and fibrosis [8]. Single‐cell analyses in liver fibrosis have revealed extensive heterogeneity and intercellular communication between hepatic stellate cells and macrophages, highlighting the importance of stromal–immune crosstalk in shaping inflammatory liver microenvironments [9]. Additional studies have demonstrated that altered adhesion signaling between activated stellate cells and neutrophils contributes to fibrotic remodeling, further emphasizing the role of intercellular communication in chronic liver injury [10]. Liver sinusoidal ECs serve as central regulators of vascular integrity and immune cell trafficking within the liver. Experimental studies in fibrotic models have shown that immune–endothelial interactions critically contribute to hepatic endothelial injury and inflammatory amplification [11]. Moreover, recent work dissects the hepatic inflammatory ecosystem of ACLF using single‐cell and spatial multi‐omics and identifies annexin A1 (ANXA1) that actively promotes inflammation resolution via the FPR1 signaling axis, representing a potential novel therapeutic target for liver failure [12]. However, the transcriptional remodeling of ECs and their communication networks with immune populations during the transition from chronic liver disease to ACLF remains poorly characterized.

The advent of single‐cell RNA sequencing (scRNA‐seq) has enabled high‐resolution mapping of cellular heterogeneity and intercellular signaling in liver diseases. Beyond ACLF, multimodal single‐cell profiling of human liver regeneration has uncovered tightly coordinated interaction networks among endothelial, stromal, and immune cells, underscoring the importance of spatially organized cellular crosstalk in hepatic injury and repair [13]. Similarly, studies in metabolic liver disease have demonstrated that modulation of the liver microenvironment critically shapes disease progression, further supporting the concept that cellular communication networks are central to liver pathology [14]. Nevertheless, a comprehensive single‐cell atlas of intrahepatic NPCs in ACLF, integrating endothelial dysfunction with immune remodeling and ligand–receptor signaling, is still lacking.

In this study, we established a murine model of ACLF that recapitulates key pathological features, including acute liver injury superimposed on chronic fibrosis and systemic inflammation. By performing scRNA‐seq of liver NPCs, we constructed a high‐resolution cellular atlas of ACLF, revealing extensive remodeling of immune and stromal compartments. We identified pronounced transcriptional reprograming of ECs toward a pro‐inflammatory state associated with mitochondrial dysfunction and activation of inflammatory signaling pathways. Ligand–receptor analysis demonstrated a global reorganization of intercellular communication, with ECs emerging as major signaling hubs. Furthermore, we delineated immune cell dynamics during ACLF progression, providing insight into coordinated programs of immune activation.

Collectively, we provide a systematic single‐cell characterization of endothelial−immune interactions in ACLF and mechanistic insight into microenvironmental imbalance during hepatic failure, highlighting intercellular communication networks as potential therapeutic targets.

## 2. Materials and Methods

### 2.1. Experimental Animals

Twenty male C57BL/6 mice (6–8‐weeks old) were purchased from Cavens Biotechnology Co., Ltd. (Changzhou, China). Mice were randomly and equally divided into the control group and the ACLF group. The ACLF phenotype was mimicked by combining long‐term CCl_4_ administration with an acute LPS/D‐GalN challenge [15]. Briefly, the ACLF group was subjected to an 8‐week regimen of CCl_4_ (0.6 mL/kg, i.p., twice per week, MCE, HY‐Y0298), while the control group received a vehicle (olive oil, MCE, HY‐108749). At 24 h post‐chronic induction, mice in the ACLF group were further challenged with a single dose of D‐GalN (1000 mg/kg, MCE, HY‐N0210) and LPS (100 ng/kg, Beyotime, ST1470). A total of 20 mice (*n* = 10 for the Control group and *n* = 10 for the ACLF group) were utilized. To optimize tissue usage, organs and serum from the same animals were multiplexed across downstream assays: serum from 5 mice per group was used for biochemical analysis, liver tissues from three mice per group were processed for histological staining (H&E, Masson, and TUNEL), and fresh liver samples from the remaining four mice per group were allocated for NPC scRNA‐seq.

### 2.2. Biochemical Analysis of Liver Function

Plasma levels of alanine aminotransferase (ALT) and aspartate aminotransferase (AST) were measured to evaluate hepatic injury using an automated biochemical analyzer (Mindray, Shenzhen, China) and the corresponding IFCC‐standardized reagent kits.

### 2.3. H&E Staining

For histological evaluation, liver specimens were processed using an automated tissue dehydrator through a graded ethanol series, cleared in xylene, and infiltrated with paraffin at 60°C. The paraffin‐embedded tissues were then sectioned into slices using a microtome (R35, Feather Safety Razor Co., Ltd., Osaka, Japan). Following mounting and baking at 60°C for 3 h, the sections underwent deparaffinization and rehydration. Nuclear staining was performed using Mayer’s hematoxylin (H9627; Sigma–Aldrich, St. Louis, MO, USA) for 5 min, followed by counterstaining with 1% aqueous eosin (71014544; Sinopharm Co., Ltd., Shanghai, China) for 5 min. Finally, the sections were dehydrated, cleared in xylene, and mounted with neutral resin for histopathological examination via light microscopy.

### 2.4. Masson’s Trichrome Staining

For the histological evaluation of hepatic fibrosis, paraffin‐embedded liver sections were prepared following the same dehydration, clearing, and infiltration procedures as described above. After deparaffinization and rehydration, the sections were processed for Masson’s trichrome staining to visualize collagen deposition. Briefly, nuclear staining was performed using Weigert’s iron hematoxylin (H9627; Sigma–Aldrich, St. Louis, MO, USA) for 5 min, followed by differentiation in 1% acid alcohol. The sections were then stained with Ponceau‐acid fuchsin (F8129; Sigma–Aldrich) for 3 min and differentiated in 1% phosphomolybdic acid (20029916; Sinopharm Chemical Reagent Co., Ltd., Shanghai, China) for 5 min. Subsequently, the sections were counterstained with aniline blue (71003644; Sinopharm Co., Ltd.) for 5 min to label collagen fibers. After a 1 min rinse in 1% glacial acetic acid, the sections were rapidly dehydrated through a graded ethanol series, cleared in xylene, and mounted with a neutral resin. Histopathological assessment and image acquisition were performed using a light microscope (BX53; Olympus, Tokyo, Japan).

### 2.5. TUNEL Assay

To detect cell apoptosis, paraffin sections were deparaffinized, rehydrated, and treated with Proteinase K (Servicebio, Wuhan, China) at 37°C for 20 min. After equilibration, the sections were incubated with a TUNEL reaction mixture (TDT enzyme, dUTP, and buffer at a 1:5:50 ratio; Servicebio) in a humidified chamber at 37°C for 1 h. Nuclei were counterstained with DAPI (Servicebio) for 10 min. Sections were then washed and mounted with an anti‐fluorescence quenching medium. Images were captured using a fluorescence microscope (IX71; Olympus, Tokyo, Japan), with apoptotic cells emitting green fluorescence and the nuclei emitting blue fluorescence.

### 2.6. Immunofluorescence Staining for Lgals9–Ighm Interaction

To verify the Lgals9–Ighm interaction, paraffin‐embedded tissue sections were prepared following the same dehydration, clearing, infiltration, embedding, sectioning, and baking procedures as described above. After deparaffinization and rehydration, antigen retrieval was performed in an EDTA buffer or citrate buffer. Endogenous peroxidase was blocked with 3% hydrogen peroxide, and non‐specific binding was blocked with 3% BSA.

Sections were first incubated with CD19 primary antibody (27949‐1‐AP; Proteintech, Rosemont, IL, USA) overnight at 4°C. After PBS washes, the sections were incubated with HRP‐conjugated secondary antibody (SA00001‐2; Proteintech, Rosemont, IL, USA), followed by TSA fluorescent dye reaction (RC0086‐45RM; Shanghai Huilan Biotechnology Co., Ltd., Shanghai, China). Antibody stripping was then performed. Next, the sections were incubated with CD31 primary antibody (28083‐1‐AP; Proteintech, Rosemont, IL, USA), followed by HRP‐conjugated secondary antibody and TSA reaction, followed by antibody stripping. This process was repeated for the Lgals9 primary antibody (17938‐1‐AP; Proteintech, Rosemont, IL, USA) and finally for the Ighm primary antibody (66484‐1‐Ig; Proteintech, Rosemont, IL, USA). After the final TSA reaction, the nuclei were counterstained with DAPI (C1002; Beyotime, Shanghai, China) for 10 min. The sections were sealed with an anti‐fade mounting medium, and images were acquired using an inverted microscope (IX71; Olympus, Tokyo, Japan) and a multi‐channel fluorescence scanner.

### 2.7. Enzyme‐Linked Immunosorbent Assay (ELISA)

Plasma IL‐6, TNF‐α, and IFN‐γ levels were determined using an ELISA kit (YB‐IL6/TNF‐α/IFN‐γ‐Mu; Shanghai Yubo Biological Technology Co., Ltd., Shanghai, China) per the manufacturer’s protocol. Briefly, plasma was isolated by centrifugation at 1000 × g for 15 min. Samples and standards were incubated in pre‐coated plates at 37°C for 60 min, followed by successive 37°C incubations with a biotinylated antibody for 60 min and Streptavidin–Biotin Complex (SABC) for 30 min. After the TMB chromogenic reaction, the absorbance at 450 nm was measured using a microplate reader (MK3; Thermo Fisher Scientific, Waltham, MA, USA). IL‐6, TNF‐α, and IFN‐γ concentrations were calculated via the standard curve.

### 2.8. Liver NPC Isolation and scRNA‐Seq

Mouse hepatic tissues were rapidly harvested and maintained on ice to ensure cell viability. The samples were washed with ice‐cold PBS to remove blood and debris and then minced into ~0.5 mm^3^ fragments. Tissue dissociation was performed using 5 mL of an enzyme cocktail containing 1 mg/mL Collagenase I, 1 mg/mL Collagenase II, and 0.5 mg/mL Neutral Protease (Sigma–Aldrich, St. Louis, MO, USA) at 37°C for 30 min with shaking at 100 rpm, following established protocols for comprehensive hepatic cell isolation [16, 17]. The reaction was neutralized with PBS containing 10% fetal bovine serum (FBS), followed by gentle pipetting. The resulting suspension was filtered through a 40 μm cell strainer and centrifuged at 500 × g for 5 min at 4°C. To ensure high cell viability, red blood cells were removed using aredd bloodcells Solution (MACS 130‐094‐183; Miltenyi Biotec, Bergisch Gladbach, Germany) for 5 min at room temperature. Dead cells were subsequently eliminated using the Dead Cell Removal Kit (MACS 130‐090‐101; Miltenyi Biotec). The purified single‐cell suspension was resuspended in PBS containing 0.04% BSA and adjusted to a concentration of 700–1200 cells/μL. Single‐cell capturing and library construction were performed using the Chromium Single Cell 3′ Reagent Kits (v3.1) on the 10× Chromium platform (10× Genomics, Pleasanton, CA, USA) with a target recovery of 8000 cells. Sequencing was conducted by LC‐Bio Technology (Hangzhou, China) on the Illumina NovaSeq 6000 system using a 150 bp paired‐end strategy.

### 2.9. Raw Data Processing

Raw sequencing data were converted to the FASTQ format using Illumina bcl2fastq (v5.0.1). Sample‐specific cell quantification and gene expression profiling were performed using the Cell Ranger pipeline (v7.2.0; 10× Genomics). Reads were aligned to the Ensembl GRCm39 reference genome to generate the gene–barcode matrix. These initial processing steps and bioinformatic analyses were conducted with assistance from LC‐Bio Technology (Hangzhou, China).

### 2.10. Data Quality Control and Preprocessing

Downstream analysis was performed using the Seurat R package (v4.4.0). To ensure data integrity, low‐quality cells with fewer than 500 detected genes or a mitochondrial transcript content exceeding 20% were excluded. Potential doublets were identified and removed using DoubletFinder (v2.0.4). Following the exclusion of low‐quality and multiplet cells, the data were normalized via the NormalizeData function, and highly variable genes were identified using the FindVariableFeatures. After data scaling with ScaleData, principal component analysis (PCA) was performed for dimensionality reduction using RunPCA. Cell clustering was subsequently conducted using FindNeighbors and FindClusters, with the resulting clusters visualized via t‐SNE or uniform manifold approximation and projection (UMAP) using the DimPlot function.

### 2.11. Ligand–Receptor Interaction Analysis

Cell–cell communication was analyzed using CellChat (v2.1.2) based on the CellChatDB.mouse database (Secreted Signaling, ECM‐receptor, and Cell–Cell Contact). Communication probabilities were calculated using the triMean method, accounting for the relative proportions of each cell group to ensure robust estimation. Significant interactions were filtered, requiring a ligand/receptor expression in ≥10% of cells per cluster and a minimum of 10 cells per group. Differential signaling was identified via permutation tests with thresholds of *p* < 0.05 and |log_2_FC| >0.05, followed by visualization using circle and bubble plots.

### 2.12. Statistical Analysis

Statistical analyses and figure generation were performed using GraphPad Prism 10.4.2. All data are expressed as the mean ± SEM. Differences between groups were analyzed using an unpaired two‐sided Student’s *t*‐test. Significance thresholds were set at *p* < 0.05, with asterisks indicating the level of significance. Single‐cell Data Analysis was performed using the OmicStudio tools created by LC‐BIO Co., Ltd. (Hangzhou, China) at https://www.omicstudio.cn/cell.

### 2.13. Generative AI Statement

The author(s) declared that generative AI was not used in the creation of this manuscript.

## 3. Results

### 3.1. Establishment and Validation of a Mouse Model for ACLF

**Figure 1:**
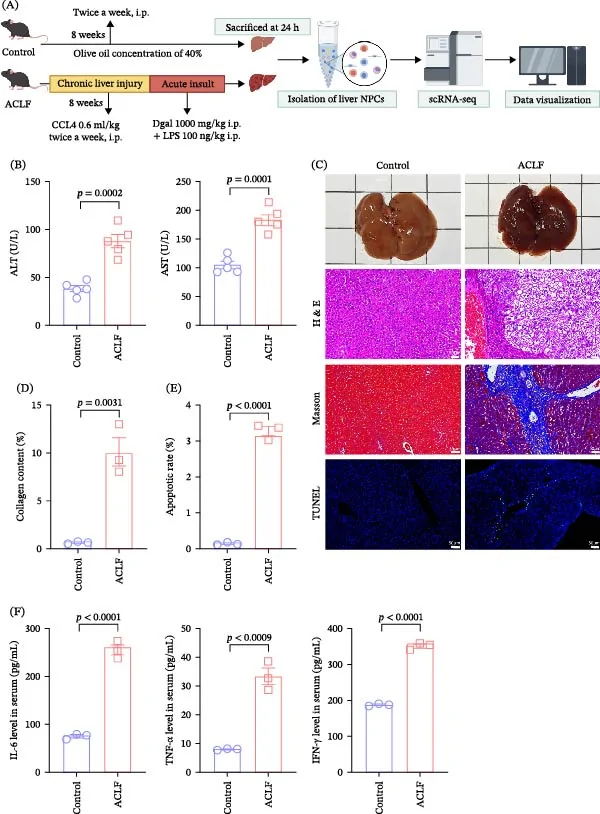
Establishment and evaluation of the ACLF mouse model. (A) Schematic workflow of the experimental design, illustrating the procedures for animal model construction, sample collection, and subsequent assays. The schematic diagram was drawn by Figdraw. (B) Plasma levels of ALT and AST in control and ACLF mice at 24 h after injury. (*n* = 5). (C) Representative histological images of liver tissues from control and ACLF groups, including H&E staining, Masson’s trichrome staining, and TUNEL staining. Scale bar, 50 μm. (*n* = 3). (D, E) Quantitative analysis of liver fibrosis based on Masson staining (D) and the number of TUNEL‐positive apoptotic cells (E) (*n* = 3). (F) Plasma levels of IL‐6, TNF‐α, and IFN‐γ. (*n* = 3). Data are presented as mean ± SEM. Statistical significance was determined using two‐sided unpaired Student’s *t*‐test, with exact *p*‐values indicated directly above each comparison (*p* < 0.05 was considered statistically significant; ns = not significant).

To investigate the underlying mechanisms of ACLF, we established an experimental animal model (Figure 1A). This model recapitulated the hallmark pathological features of clinical ACLF, characterized by profound dysfunction and a systemic inflammatory response.

Biochemical analysis revealed that plasma ALT and AST levels were markedly elevated in the ACLF group compared to the control group at 24 h after acute injury, indicating acute and severe hepatocellular damage (Figure 1B). It is worth noting that the serum ALT and AST levels measured at 24 h in our model were lower than those reported at 6 h by Tang et al. [15]. This variance is consistent with the known time‐dependent clearance kinetics of transaminases, which typically peak during the early acute phase and progressively decline due to rapid in vivo turnover [18, 19]. Histological examination further corroborated these findings (Figure 1C, Supporting Information 1: Figure S1). H&E staining showed that while the control group maintained intact lobular architecture and orderly arranged hepatocytes without obvious inflammatory infiltration, the ACLF group exhibited significant parenchymal loosening, increased vacuolated areas, and massive inflammatory cell infiltration (Figure 1C, Supporting Information 1: Figure S2). Furthermore, Masson’s trichrome staining demonstrated a substantial increase in collagen fiber deposition in the ACLF group relative to the controls, confirming the presence of underlying chronic hepatic fibrosis (Figure 1C,D).

To assess the extent of hepatic cell death, we performed TUNEL staining, which revealed a statistically significant increase in the number of apoptotic cells within the hepatic tissues of ACLF mice compared to the control group (Figure 1C,E). Given that ACLF is clinically associated with a “cytokine storm,” we further evaluated the systemic inflammatory response. Plasma levels of key pro‐inflammatory cytokines, including IL‐6, TNF‐α, and IFN‐γ, were significantly upregulated in ACLF mice (Figure 1F), reflecting a state of heightened systemic inflammation. Collectively, these data demonstrate that our model reproduces the core pathological components of ACLF, including acute hepatic insult superimposed on chronic fibrosis, extensive apoptosis, and a systemic inflammatory response, thereby providing a robust experimental platform for subsequent mechanistic dissections.

### 3.2. Single‐Cell Transcriptomic Landscape of the NPC Compartment in ACLF Livers

To dissect the cellular heterogeneity and molecular remodeling during ACLF progression, we performed scRNA‐seq on NPCs isolated from control and ACLF mouse livers. Following stringent quality control and cell filtering, the remaining cells exhibited high‐quality transcriptomic profiles, with consistent gene detection, UMI counts, and low mitochondrial content across samples (Figure 2A). 13,068 high‐quality cells were successfully obtained in the control group and 19,333 cells in the ACLF group. Unsupervised clustering and UMAP analysis identified 30 distinct cell clusters (Figure 2B).

Figure 2Single‐cell transcriptomic landscape of non‐parenchymal cells (NPCs) in control and ACLF mouse livers. (A) Quality control metrics of single‐cell RNA sequencing data across all samples after cell filtering. The figure displays the distribution of detected gene counts (left), total UMI counts (center), and the percentage of mitochondrial gene expression (right) per cell. (B) UMAP visualization of 30 major cell clusters, with each color representing a distinct annotated cell population. (C) UMAP projection of integrated datasets showing the distribution of cells derived from control and ACLF liver tissues. (D) Heatmap illustrating the relative expression of main marker genes across the identified cell clusters. (E) UMAP plot highlighting the major liver cell populations identified. HSC, hepatic stellate cell. (F) Bar plot showing the relative proportion of control (blue) and ACLF (red) cells within each cell population. (G) Dot plot displaying the expression levels of key inflammatory genes between control and ACLF groups (*n* = 4 mice per group). Data are presented as mean ± SEM. Statistical significance was determined using two‐sample Student’s *t*‐test with Benjamini–Hochberg adjustment for multiple comparisons. Genes with significant upregulation in the ACLF group are indicated with asterisks on the axis (adj *p* < 0.01). Data are derived from single‐cell RNA sequencing experiments (*n* = 4).

**Figure:**
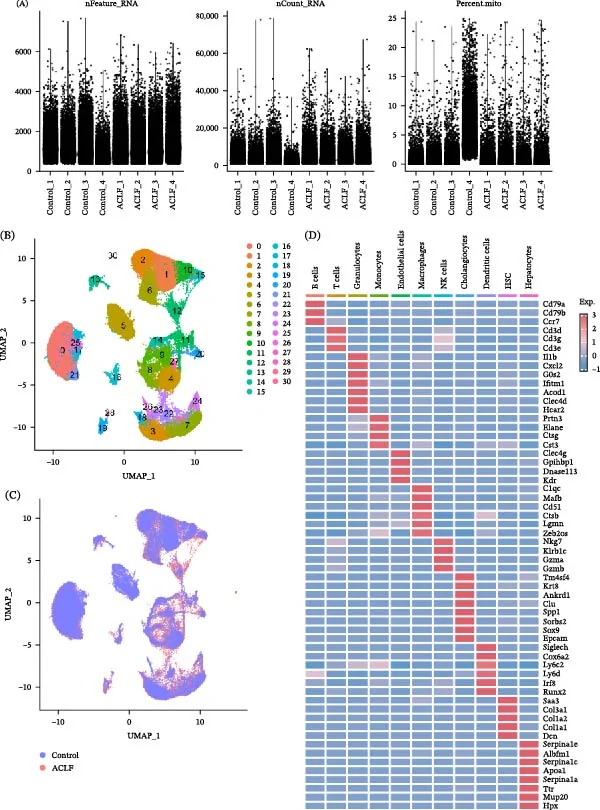

**Figure:**
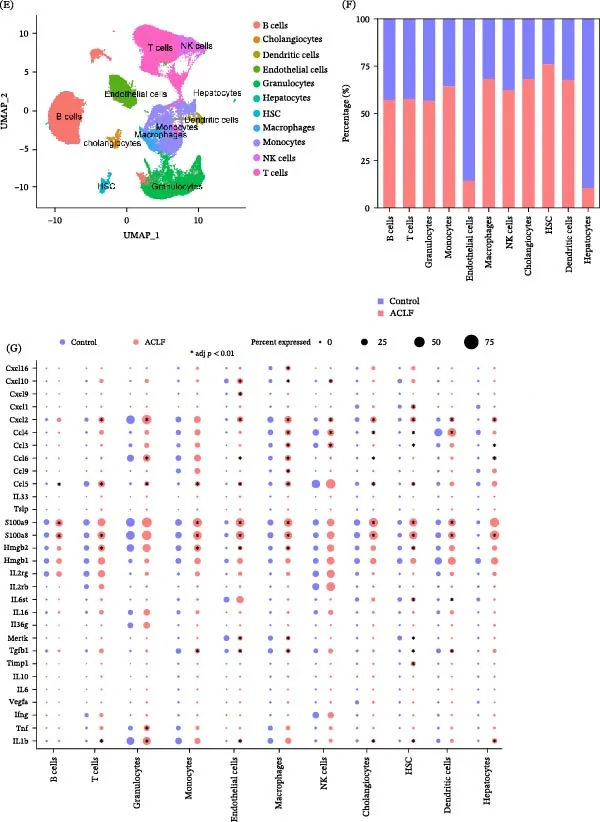

Integration of the datasets revealed that while all major cell populations were present in both groups, their distributions shifted significantly upon ACLF induction (Figure 2C). The identities of these clusters were validated by the expression of lineage‐specific marker genes, B cells (*Cd79a* and *Cd79b*), T cells (*Cd3d*, *Cd3e*, and *Cd3g*), granulocytes (*Il1b*, *Cxcl2*, and *G0s2*), monocytes (*Prtn3* and *Ctsg*), ECs (Clec4g, Gpihbp1, and Kdr), macrophages (*Mafb*, *Cd5l*, and *Ctsb*), NK cells (*Nkg7*, *Klrb1c*, and *Gzma*), cholangiocytes (*Tm4fs4*, *Krt8*, and *Ankrd1*), DCs (*Siglech*, *Cox6a2*, and *Ly6c2*), HSC (*Saa3*, *Col3a1*, and *Dcn*), and hepatocytes (*Albfm1*, *Apoa1*, and *Serpina1e*), as illustrated by the heatmap (Figure 2D), and were further annotated into major cell lineages based on canonical markers (Figure 2E). Notably, we observed a dramatic reorganization of the hepatic immune and stromal compartments. Quantitative analysis of NPC proportions revealed that the relative proportion of ECs was significantly reduced in ACLF livers compared to controls (*p* < 0.05). Although mean proportions of immune subsets, including B cells, T cells, granulocytes, monocytes, macrophages, and NK cells, exhibited numerical increases in the ACLF state, these differences did not reach statistical significance (Figure 2F, Supporting Information 1: Figure S3).

To systematically evaluate the transcriptional alterations in hepatic NPCs during ACLF progression, we analyzed the expression profiles of key immune‐related genes using a dot plot (Figure 2G). Differential expression analysis using two‐sample *t*‐tests revealed that genes associated with inflammation and leukocyte chemotaxis, including *Cxcl10*, *Cxcl2*, *Ccl4*, *Ccl6*, *Ccl5*, *S100a9*, *S100a8*, *Hmgb2*, *Mertk*, and *Tgfb1*, were significantly upregulated in the ACLF group compared to the control group (adjusted). This transcriptomic shift underscores a coordinated inflammatory response across the entire NPC landscape. Collectively, these findings provide a comprehensive single‐cell atlas of the ACLF liver, identifying the cellular drivers of the inflammatory surge and establishing a foundation for high‐resolution dissection of cell‐type‐specific contributions to the disease pathogenesis.

### 3.3. ACLF Induces Profound Sub‐Population Remodeling and Transcriptomic Shifts in Hepatic ECs

Given the pivotal role of the hepatic vasculature in maintaining liver homeostasis, we performed high‐resolution re‐clustering of the EC compartment (Figure 3A). This analysis identified seven distinct EC sub‐populations characterized by unique transcriptional signatures (Figure 3B). The identity of each sub‐cluster was defined by the most significantly differentially expressed marker genes (Figure 3C) and further validated using canonical marker profiles (Figure 3D). Supported by canonical marker expression and established reference literature [20, 21], this analysis identified eight defined EC subtypes: periportal liver sinusoidal ECs (PPLSEC), pericentral liver sinusoidal ECs (PCLSEC), Rspo3^+^ ECs, Ebf1^+^ ECs, Skap1^+^ ECs, Lyz2^+^ ECs, microvascular ECs (MVEC), and mid‐lobular ECs (MLEC) (Figure 3D).

Figure 3Heterogeneity and transcriptional alterations of liver endothelial cells in ACLF. (A) UMAP projection showing the integrated clustering of all endothelial cells (ECs) from control and ACLF mouse livers. (B) UMAP visualization of EC sub‐clusters, revealing seven distinct second‐level sub‐populations. (C) Heatmap illustrating the top 10 differentially expressed marker genes across the seven EC sub‐clusters. (D) Dot plot displaying the expression of canonical marker genes across the eight EC sub‐clusters. Dot size indicates the percentage of cells expressing the gene, and color intensity represents the average expression level. (E) Bar plot showing the relative proportions of control (blue) and ACLF (red) cells within each EC sub‐cluster. (F) Volcano plot displaying differentially expressed genes (DEGs) in Lyz2^+^ sub‐clsuster between the control and ACLF groups. Dark red and dark blue dots represent significantly up‐ and down‐regulated genes, respectively (|log_2_FC| >0.05, *p* < 0.05). (G) Bar plot of representative GSVA terms enriched in the Lyz2^+^ sub‐clsuster control and ACLF endothelial cells. Data are derived from single‐cell RNA sequencing experiments (*n* = 4). Data in (E) are presented as mean ± SEM. Statistical significance was calculated using two‐tailed unpaired Student’s *t*‐test, with exact *p*‐values indicated directly above each comparison in (E) (*p* < 0.05 was considered statistically significant, ns = not significant).

**Figure:**
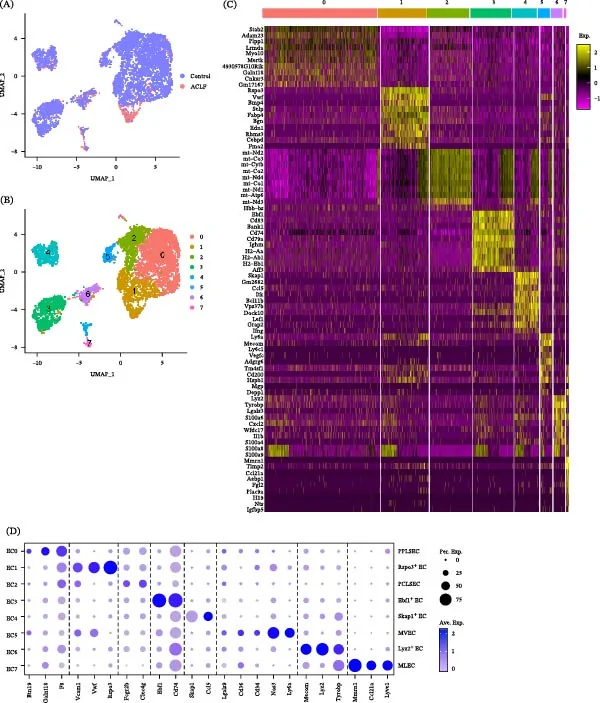

**Figure:**
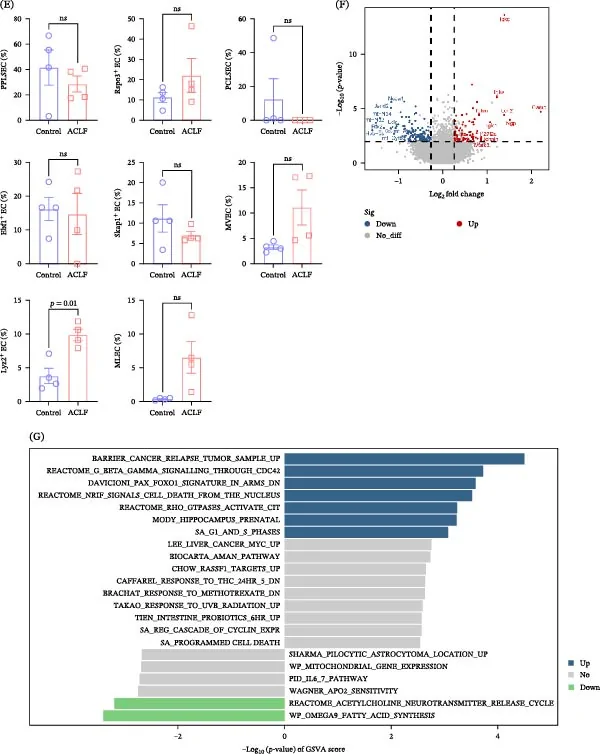

Notably, we observed a striking redistribution of EC sub‐populations following ACLF induction (Figure 3E). Quantitative assessment revealed marked alterations in the relative proportions of EC sub‐clusters between control and ACLF groups, with Lyz2^+^ ECs exhibiting a distinct shift. To further characterize the molecular response of Lyz2^+^ ECs to the ACLF‐associated inflammatory microenvironment, we identified differentially expressed genes (DEGs) in this sub‐cluster between the two groups (Figure 3F). The volcano plot highlighted a dramatic transcriptomic shift in Lyz2^+^ ECs, characterized by the significant upregulation of key immune activation components and inflammatory/antimicrobial markers, including *Igkc*, *Igha*, *Ighm*, *Camp*, *Ngp*, and *Lcn2* (Figure 3F). Functional enrichment analysis via gene set variation analysis (GSVA) further elucidated the biological implications of these transcriptional alterations in Lyz2^+^ ECs (Figure 3G). Specifically, Lyz2^+^ ECs from the ACLF group exhibited marked upregulation of pathways related to cell cycle progression and nuclear cell death signals (e.g., SA G1 AND S PHASES, NRIF SIGNALS CELL DEATH FROM THE NUCLEUS), as well as Rho GTPase/CDC42 signaling cascades (RHO GTPASES ACTIVATE CIT, G BETA GAMMA SIGNALLING THROUGH CDC42). Conversely, pathways governing essential metabolic processes (e.g., OMEGA9 FATTY ACID SYNTHESIS) and homeostatic signaling (ACETYLCHOLINE NEUROTRANSMITTER RELEASE CYCLE) were significantly downregulated (Figure 3G). Collectively, these findings indicate that ACLF triggers a comprehensive transition of hepatic ECs particularly the Lyz2^+^ EC sub‐cluster from a homeostatic state toward a dysfunctional, pro‐inflammatory, and metabolically impaired phenotype.

### 3.4. ACLF Induces Systemic Rewiring of the Hepatic Intercellular Communication Network

To systematically characterize the evolution of the hepatic microenvironment during the progression of ACLF, we performed ligand‐receptor interaction analysis to map the intercellular communication landscape. Global quantitative analysis revealed a profound reorganization of cellular crosstalk in the ACLF state; the total number of inferred interactions surged from 888 in the control group to 1134, reflecting a marked increase in communication frequency under pathological conditions. However, the overall interaction strength exhibited a marginal decline from 0.116 to 0.104, suggesting that while the volume of cellular “dialogue” expanded, the average signaling potency was somewhat attenuated, likely due to the emergence of numerous new, but weaker, signaling connections (Figure 4A).

Figure 4Comparative analysis of the intercellular communication landscape and EC‐B cell in control and ACLF. (A) Global quantification of intercellular communication in control and ACLF groups. Left: Total number of interactions; Right: Overall interaction strength. (B) Heatmap illustrating the differential number of ligand‐receptor pairs across all cell subpopulations. Blue shading indicates increased signaling intensity in the control group, while red shading denotes enriched communication within the ACLF group. (C) Differential network plot visualizing shifts in communication strength between control and ACLF (model) groups. Blue edges represent signaling pathways predominant in the control group, whereas red edges highlight enhanced communication in the ACLF group. Edge thickness is proportional to the magnitude of the change in communication strength. (D, E) Dot plots showing the most significantly upregulated (D) and downregulated (E) ligand–receptor pairs specifically between EC subsets and B cell subpopulations in ACLF compared to controls. The x‐axis represents specific cell–cell interaction pairs (source‐target), and the y‐axis indicates ligand–receptor pairs. Dot color reflects the communication probability, and dot size represents statistical significance (*p*‐value). (F) Bar plot showing the relative contribution of ligand–receptor pairs to the GALECTIN signaling pathway. (G) Heatmap showing the overall communication probability of the GALECTIN signaling network between EC subsets and B cell subpopulations. Color gradient represents the communication probability. (H) CellChat network analysis illustrating autocrine and paracrine GALECTIN signaling interactions between EC subsets (left nodes, sources) and B cell subpopulations (right nodes, targets) in Control (left) and ACLF (right) groups. Each color represents a distinct cell subpopulation. Solid circles denote signal sources (senders) and hollow circles denote signal targets (receivers). Line thickness corresponds to interaction strength. Self‐connecting loops indicate autocrine signaling, whereas edges extending to multiple distinct cell subsets represent paracrine transmission. Data are derived from single‐cell RNA sequencing analysis (*n* = 4).

**Figure:**
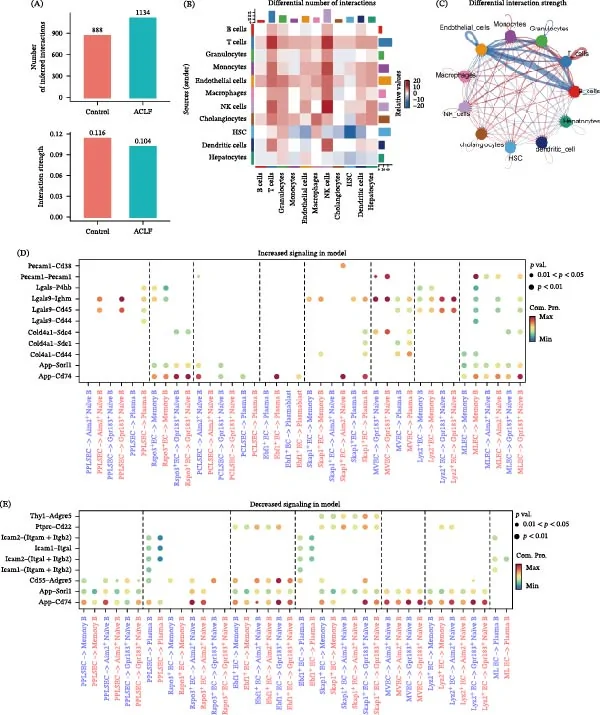

**Figure:**
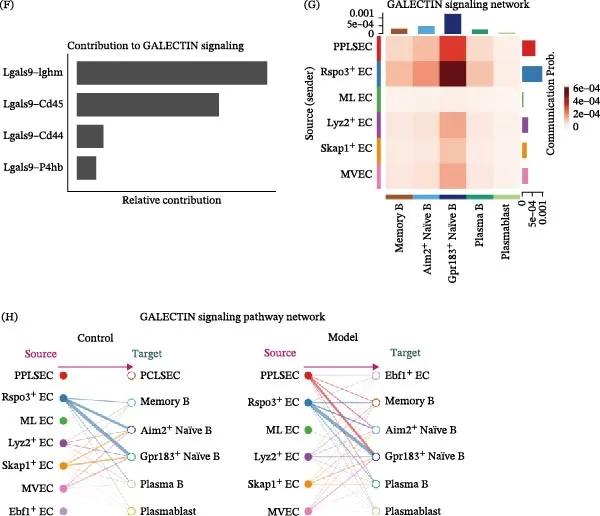

Through integrated analysis of the differential heatmap (Figure 4B) and differential network plot (Figure 4C), we identified the core cell populations driving this signaling surge. Our results revealed that B cells, T cells, ECs, NK cells, macrophages, and granulocytes emerged as the cell types with the most prominent signaling enrichment in the ACLF group. The evolution of signaling networks from the control to the ACLF state demonstrates that ACLF triggers a fundamental transition in the hepatic communication machinery: shifting from physiological homeostasis toward a pro‐inflammatory hub centered on ECs and B cells.

Given these global shifts, we performed targeted interactome analysis across the 8 EC subtypes and 5B cell subpopulations to decipher subtype‐specific signaling dynamics. At the molecular level, dot plot analysis revealed significant upregulation of pro‐inflammatory and immune activation axes in the ACLF group (Figure 4D). In support of this finding, UMAP feature plots confirmed the expression pattern of Lgals9 across the eight distinct EC subclusters (Supporting Information 1: Figure S4).

Specifically, Lgals9‐mediated interactions predominantly Lgals9–Ighm and Lgals9–Cd45 exhibited exceptionally high communication probabilities and statistical significance (*p* < 0.01) across multiple EC‐to‐B‐cell channels, particularly from PPLSEC, Rspo3^+^ EC, Lyz2^+^ EC, and MVEC to Gpr183^+^ naïve B and plasma B cells. Conversely, ACLF induction triggered a profound and widespread suppression of key protective and homeostatic regulatory axes between ECs and B cells (Figure 4E). The Cd55–Adgre5 interaction, essential for restricting complement‐mediated tissue injury and supporting cell adhesion, was significantly attenuated. Furthermore, communication probabilities for amyloid precursor protein (APP)‐related pairs (App–Sorl1 and App–Cd74) as well as adhesion molecules such as Icam2–(Itgam+Itgb2) experienced notable declines in specific sub‐cluster interactions. Comparative pathway evaluation further identified GALECTIN signaling as a dominant pathway, with Lgals9–Ighm and Lgals9–Cd45 providing the highest relative contributions (Figure 4F). Heatmap analysis demonstrated that GALECTIN signaling probability was profoundly enriched, with Rspo3^+^ ECs and PPLSECs acting as primary signal senders broadly targeting B‐cell subsets, among which Gpr183^+^ naïve B cells represented the most prominent receiver (Figure 4G). CellChat network mapping corroborated a substantial intensification of GALECTIN signaling in the ACLF group compared to controls, characterized by denser and stronger paracrine interactions extending from multiple EC sub‐clusters (e.g., PPLSEC, Rspo3^+^ EC, and Lyz2^+^ EC) to diverse B‐cell subpopulations. GALECTIN pathway network analysis revealed that the cellular composition regulating the GALECTIN function differs between groups. Specifically, the control group was mainly dominated by interactions between Rspo3^+^ EC cells (ligands) and Aim2^+^/Gpr183^+^ naïve B cells and memory B cells (receptors), whereas the ACLF group was mainly dominated by interactions between PPLSEC and Rspo3^+^ EC cells (ligands) and Gpr183^+^ naïve B cells and memory B cells (receptors) (Figure 4H). Together, these findings delineate a dual pathological mechanism during ACLF progression, where the robust gain of endothelial‐driven pro‐inflammatory signaling (via GALECTIN axes) operates in tandem with the active loss of endogenous immunometabolic and tissue‐protective barriers.

### 3.5. Lineage Heterogeneity and Transcriptomic Signatures of Hepatic B Cells in ACLF

**Figure 5:**
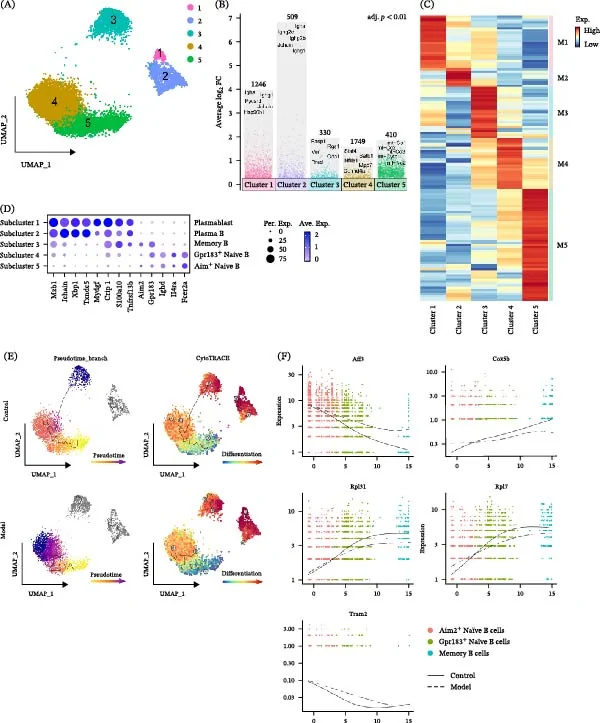
Single‐cell transcriptomic landscape and developmental trajectories of B cells in ACLF. (A) UMAP visualization highlighting five transcriptionally distinct B‐cell subpopulations. (B) Volcano plots showing cluster‐specific marker genes. The y‐axis represents the log_2_ fold change of average gene expression in the target cluster versus all other clusters. Points are colored by adjusted *p*‐values, with the top five upregulated genes annotated. (C) Heatmap of the top 10 differentially expressed marker genes defining each B cell subcluster. (D) Dot plot illustrating the expression of canonical marker genes used for the identification of the five B‐cell sub‐clusters. Dot size indicates the percentage of cells expressing the gene, and color intensity represents the average expression level. (E) Pseudotime trajectory branching and CytoTRACE analysis comparing control and ACLF groups. (F) Kinetic expression profiles of the pseudotime‐dependent genes. Each point represents an individual cell, plotted by pseudotime (x‐axis) and normalized expression level (y‐axis). Data are derived from single‐cell RNA sequencing analysis (*n* = 4).

To further elucidate the B cell expansion observed in our global atlas, we performed high‐resolution re‐clustering of the hepatic B cell compartment (Figure 5A). Volcano plot analysis defined the cluster‐specific marker genes for each subpopulation, highlighting key molecules involved in B cell activation, maturation, and effector functions (Figure 5B). The transcriptomic signatures of these five sub‐clusters were further established by heatmap analysis of top differentially expressed marker genes (Figure 5C, Supporting Information 2: Table S1). Supported by canonical marker expression profiles and established literature [22], this analysis delineated five transcriptionally distinct B cell subpopulations: Plasmablast, Plasma B, Memory B, Gpr183^+^ naïve B, and Aim2^+^ naïve B cells and validated their distinct lineage identities via canonical marker dot plot visualization (Figure 5D).

To decipher the ontogenic relationships and differentiation dynamics among these subpopulations, we reconstructed their developmental continuum using pseudotime trajectory and CytoTRACE analyses (Figure 5E). Trajectory branching and CytoTRACE scoring revealed a clear evolutionary progression originating from naïve/undifferentiated progenitor‐like states and bifurcating toward distinct terminal effector states, illustrating an orchestrated cell fate decision process within the liver during ACLF progression. Among them, the differentiation trajectories of memory B cells in the control and ACLF groups were distinctly different, manifested as a continuous differentiation relationship between memory B cells and naive B cells in the control group, whereas such a continuous differentiation relationship was absent in the ACLF group, suggesting that the memory B cell population in the ACLF group tends to be more mature in differentiation, with a differentiation state close to plasma cells (Figure 5E). Transitions along the developmental axis were driven by the kinetic expression of key pseudotime‐dependent regulatory genes (Figure 5F). Temporal expression profiling revealed the dynamic induction or repression of specific driver transcripts as cells progressed along the differentiation continuum. Comparing the changes in driver gene expression during and memory B cell differentiation between the control and ACLF groups, it was found that memory B cells in the ACLF group exhibited higher expression levels of Aff3 and Cox5b and lower expression levels of Rpl31. This further confirms that memory B cells in the control and ACLF groups are in distinct states, suggesting that their functional characteristics may differ. However, specific functions still need to be confirmed by direct functional experiments (Figure 5F). To further decipher the functional heterogeneity and metabolic/immune states of hepatic B cells, we performed GSVA across the five identified B‐cell subpopulations, which revealed distinct pathway enrichment profiles in ACLF livers (Supporting Information 1: Figure S5). Collectively, these findings provide a comprehensive single‐cell resolution map of hepatic B‐cell heterogeneity, developmental trajectories, and transcriptional programs governing B‐cell fate decisions in failing livers.

### 3.6. In Situ Validation Confirms the Accumulation and Spatial Co‐Localization of LGALS9 and IgM in ACLF Liver Tissues

**Figure 6:**
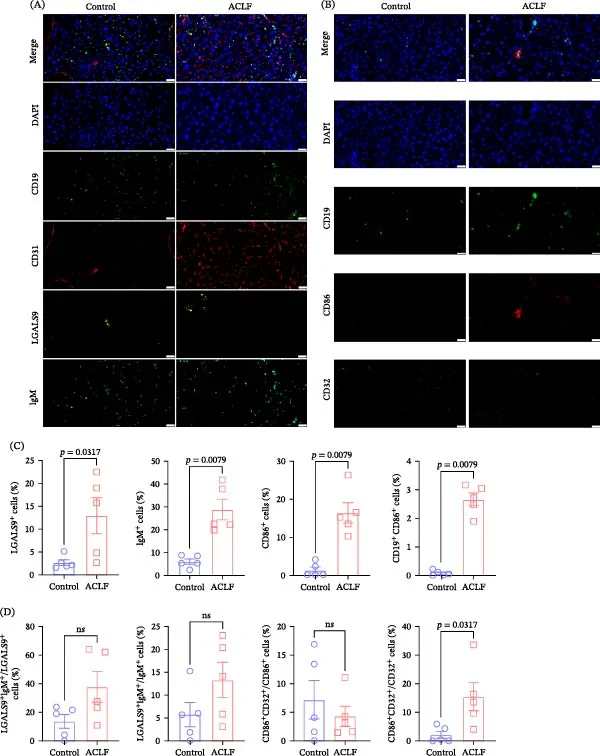
Enhanced expression and spatial co‐localization of LGALS9 and IgM in ACLF liver tissues. (A) Representative multiplex immunofluorescence images of liver tissues from the control and ACLF groups. Distinct marker expressions are visualized by staining for CD19 (green), CD31 (red), LGALS9 (yellow), IgM (cyan), and DAPI (blue). (B) Representative immunofluorescence images of CD19 (green), CD86 (red), CD32 (yellow), and DAPI (blue) in liver tissues from the control and ACLF groups. Scale bar, 25 μm. (C) Quantitative analysis of the percentage of positive cells for indicated markers (calculated as the number of specific marker‐positive cells divided by total DAPI^+^nuclei per field) between control (blue) and ACLF (red) groups. (D) Quantitative analysis of co‐localized cell percentages (calculated as the number of double‐positive co‐expressing cells divided by the total number of a specific single‐positive cell population) between control and ACLF groups. Each dot represents an individual biological replicate (*n* = 5), calculated as the mean value of three random fields per section. Data are presented as mean ± SEM. Statistical significance was determined using two‐tailed unpaired Student’s *t*‐test, with exact *p*‐values (or ns = not significant) indicated directly above each column.

To validate the critical intercellular communication axes predicted by our ligand–receptor analysis (Figure 4), we performed multiplex immunofluorescence staining on murine liver sections to evaluate the in situ expression and spatial distribution of these target molecules (Figure 6). Strikingly consistent with the prediction identifying the Lgals9–Ighm axis as a hallmark signature of ACLF, immunofluorescence profiling revealed a profound pro‐inflammatory shift in the ACLF group compared to the control group (Figure 6A). In normal control liver tissues, the LGALS9 signal remained quiescent, accompanied by only a sparse distribution of IgM^+^ cells, reflecting basal physiological homeostasis. Conversely, in ACLF tissues, LGALS9 expression was upregulated, predominantly concentrating within microenvironments characterized by intense inflammatory cell infiltration, as evidenced by dense DAPI counterstaining. Notably, merged images displayed a pronounced spatial co‐localization between high LGALS9 expression and enriched IgM signals, providing direct histological evidence for the computationally inferred interactome. To ensure a rigorous evaluation, these observations were further substantiated by quantitative analyses. The percentages of LGALS9^+^, IgM^+^, CD86^+^, and CD19^+^CD86^+^ cells were significantly elevated in the ACLF group (Figure 6C). Although the LGALS9/IgM colocalization rate showed no significant difference between the two groups (Figure 6D), the overall expansion of both cell populations firmly supports the enhanced activation of the Lgals9–Ighm axis. These findings were further corroborated by comprehensive quantifications of DAPI‐based total cell counts and positive cell populations in the support data (Supporting Information 1: Figures S6–S8).

Our single‐cell communication network previously highlighted B cells and ECs as the core driving forces propagating this pathological signaling surge. This framework was further substantiated by our histological findings, which demonstrated a marked increase in the infiltration of CD19^+^ B cells within the ACLF liver parenchyma, suggesting that the elevation of local IgM levels originates from the regional recruitment and clustering of activated B cells (Figure 6A). Furthermore, the enhanced yet structurally disrupted CD31 staining in the ACLF group highlighted conspicuous inflammation‐associated vascular and sinusoidal remodeling, which likely facilitates the endothelial adhesion and transendothelial migration of peripheral immune cells. To further define the functional phenotype of these infiltrating B cells, we evaluated the expression of the costimulatory marker CD86 and the inhibitory receptor CD32 (Figure 6B). While CD32 expression remained largely unaltered, a significant increase in CD86 signaling was observed that aligned precisely with the clustered CD19^+^ B cells (further quantified in Supporting Information 1: Figures S7 and S8), providing definitive evidence of a highly active, pro‐inflammatory B‐cell state. CD86 signaling increases that aligned precisely with the clustered CD19^+^ B cells, providing definitive evidence of a highly active, pro‐inflammatory B‐cell state. Collectively, these in situ imaging data corroborate our single‐cell network findings, demonstrating that during ACLF progression, LGALS9 and IgM‐mediated humoral immunity synergistically orchestrate an active inflammatory microenvironment superimposed on endothelial activation.

## 4. Discussion

In this study, we established an ACLF mouse model characterized by chronic fibrosis with superimposed acute hepatic decompensation, extensive hepatocyte apoptosis, and systemic inflammation. By performing scRNA‐seq on liver NPCs, we constructed a high‐resolution atlas of NPCs in ACLF livers. We found that hepatic ECs undergo a critical transcriptional shift from a homeostatic to a dysfunctional, pro‐inflammatory phenotype, marked by the suppression of mitochondrial genes and activation of NF‐κB and MAPK signaling pathways. Intercellular interactome analysis reveals that dysfunctional EC subsets—particularly Rspo3^+^ ECs and PPLSECs, act as a central vascular hub driving microenvironmental remodeling. This is mediated through the robust gain of GALECTIN/Lgals9 signaling (predominantly via the Lgals9–Ighm and Lgals9–Cd45 axes) targeting Gpr183^+^ naïve B and plasma B cells, operating in tandem with the concurrent collapse of endogenous homeostatic barriers (including the Cd55–Adgre5 and App‐related pathways). Collectively, our study delineates the endothelium–B‐cell immune communication landscape in ACLF, highlighting the Lgals9–Ighm axis as a potential key regulatory node and therapeutic target.

Systemic inflammation is increasingly recognized as a central driver of organ failure and mortality in ACLF [5, 23]. Engelmann et al. [23] comprehensively reviewed key features of systemic inflammation in acutely decompensated cirrhosis and ACLF, highlighting that elevated white blood cell counts and increased circulating inflammatory mediators are hallmarks of this syndrome. Consistent with these clinical observations, our ACLF mouse model exhibited markedly elevated plasma levels of IL‐6, TNF‐α, and IFN‐γ, and widespread upregulation of pro‐inflammatory cytokines (Il1b, Tnf, and Il6), alarmins (S100a8 and S100a9), and chemokines (Ccl5, Cxcl1, and Cxcl2) across multiple NPC populations. Our single‐cell transcriptomic data further revealed that this systemic inflammatory state is reflected within the liver as extensive immune activation, with a significant expansion of B cells, T cells, granulocytes, monocytes, macrophages, and NK cells in ACLF livers. Beyond inflammation, recent studies have also highlighted the role of immunosuppression in ACLF, including immune exhaustion and immune paralysis, which predispose patients to secondary infections and re‐escalation of organ dysfunction [23–26]. Intriguingly, recent high‐resolution single‐cell studies on human ACLF have begun to unveil unique compensatory immunoregulatory cell populations within this chaotic milieu; for instance, Xu et al. [27] identified a distinct RETN^+^ monocyte subset selectively expanded in the circulation and liver of HBV‐ACLF patients, which exerts critical anti‐inflammatory and anti‐apoptotic functions via resistin‐mediated PI3K/AKT signaling. The expansion and altered functional states of B‐cell subsets observed in our ACLF livers may reflect such immune dysregulation, warranting further investigation into the balance between immune activation and suppression in the liver.

ECs, particularly hepatic sinusoidal ECs, are critical for maintaining hepatic vascular integrity, metabolic homeostasis, and immune cell responses [28, 29]. During chronic liver disease, sinusoidal ECs progressively become dysfunctional, directly contributing to fibrosis progression, portal hypertension, and ultimately cirrhosis [28]. In the context of ACLF, the liver sinusoid has been proposed as a key battlefield where interactions among hepatocytes, ECs, hepatic stellate cells, and circulating immune cells determine disease outcomes [3]. This study characterized EC dysfunction in ACLF at a single‐cell resolution. We identified seven distinct endothelial subclusters, with one cluster (E7) significantly expanded under ACLF conditions. Transcriptomic analysis revealed a striking shift: marked upregulation of pro‐inflammatory genes and immune activation markers, accompanied by coordinated suppression of multiple mitochondrially encoded genes, suggesting severe metabolic dysfunction and mitochondrial stress. Functional enrichment analysis further confirmed the activation of NF‐κB, MAPK, chemokine, NOD‐like receptor, and IL‐17 signaling pathways in ACLF‐derived ECs. These findings closely parallel the disrupted liver inflammation ecosystem decoded in clinical ACLF. Specifically, single‐cell and spatial transcriptomic analyses of human ACLF livers by Yu et al. [12] demonstrated that massive hepatic infiltration of CCL4^+^ neutrophil subsets actively shapes a highly volatile pro‐inflammatory niche by recruiting CD14^+^S100A9^+^ inflammatory monocytes. Moreover, a recent study on hepatic lymphatic ECs in HBV‐ACLF patients demonstrated that ACLF is associated with reduced lymphatic vessel density and lymphatic EC dysfunction, mediated at least in part by SPP1 released from infiltrating monocytes/macrophages [30].

A key finding of our study is the identification of the Lgals9–Ighm axis as a prominent cell–cell communication pathway potentially involved in endothelium–immune crosstalk in ACLF. Galectin‐9, a carbohydrate‐binding lectin of the galectin family, has emerged as a critical regulator of immune responses, with its expression levels correlating with the severity of various inflammatory diseases [31]. Notably, galectin‐9 binds to the IgM B‐cell receptor (IgM‐BCR) and has been shown to modulate B‐cell signaling by coalescing pre‐existing IgM‐BCR nanoclusters, trapping the receptor, and repositioning it with inhibitory molecules including CD45 and CD22 [32]. This mechanism suggests that galectin‐9 exerts context‐dependent immunomodulatory functions, potentially either stimulating or suppressing immune responses depending on the cellular environment [31]. In our ACLF model, we observed that ECs were the primary signaling hub expressing Lgals9, which interacted with Ighm, Cd45, and Cd44 on B cells and other immune cells. This axis exhibited a high communication probability in ACLF, suggesting that Lgals9–Ighm signaling may contribute to the recruitment, retention, or activation of B cells in the inflamed liver. Beyond B cells, galectin‐9 has also been shown to differentially regulate T‐cell populations, exerting distinct functions in CD4^+^ and CD8^+^ T‐cell responses, further emphasizing its pleiotropic roles in immune modulation [33]. Collectively, these findings suggest that the Lgals9–Ighm axis may be a potential therapeutic node in ACLF; however, it should be noted that we have only validated the colocalization of molecules in this axis by immunofluorescence, and its causal role requires functional validation through genetic or pharmacological interventions.

In addition to activation of the above pro‐inflammatory pathways, we also observed marked suppression of homeostatic protective signals, particularly the Cd55–Adgre5 and App–Sorl1/Cd74 pathways, revealing another critical dimension of hepatic microenvironment collapse in ACLF. CD55, a complement regulatory protein that inhibits C3 convertase, mediates a protective “brake” that is significantly lost in ACLF, potentially leading to uncontrolled complement activation and exacerbation of liver injury and systemic inflammation [34]; impaired hepatic acute‐phase protein synthesis [35], loss of SORL1‐mediated protective signals [36], and dysregulation of the MIF/CD74 signaling axis, which has been implicated in inflammatory responses in autoimmune liver diseases and is closely associated with prognosis in acutely decompensated cirrhosis [37, 38], may all compromise the liver’s innate immune defense capabilities. The coordinated collapse of these homeostatic signals implies the failure of complement‐mediated tissue protection and acute‐phase immune defense mechanisms, likely serving as important drivers of uncontrolled inflammatory amplification and persistent liver tissue damage in ACLF [34, 35].

The APP signaling network is an emerging frontier in liver biology. APP and its proteolytic product β‐amyloid (Aβ), traditionally studied in the context of Alzheimer’s disease, have recently been established as critical regulators of liver homeostasis and fibrosis [39, 40]. The landmark study by Buniatian et al. [39] demonstrated that the hepatic Aβ content dramatically declines in human and rodent cirrhosis; Aβ deficiency (via APP knockout or antibody‐mediated depletion) upregulates classical markers of fibrosis, activates TGFβ signaling, and promotes inflammation, whereas systemic or intrahepatic elevation of Aβ42 protects the liver from CCl_4_‐induced injury. Gross et al. [40] further showed that reduced hepatic Aβ42 levels in metabolic dysfunction‐associated steatotic liver disease (MASLD) patients result from enhanced non‐amyloidogenic processing of APP. Guo et al. [41] reported that APP promotes MASH progression by upregulating death receptor 6‐mediated hepatocyte apoptosis. These observations align with our findings: in ACLF, App–Sorl1 and App–Cd74 interactions are sharply downregulated, suggesting that loss of APP‐mediated homeostatic signaling during the transition from chronic liver disease to ACLF may contribute to the functional collapse of the hepatic microenvironment. Furthermore, dysregulation of the MIF/CD74 axis plays a critical role in acutely decompensated cirrhosis—the balance between serum MIF and its soluble receptor sCD74 independently predicts the patient prognosis [38]. Together, suppression of protective mechanisms such as CD55 and loss of APP‐mediated homeostatic signaling reveal a broader principle: the pathogenesis of ACLF involves not only the gain of pro‐inflammatory signals but also active loss of endogenous protective mechanisms. This concept aligns perfectly with the systemic immunometabolic landscape observed in human cohorts; Feio‐Azevedo et al. [42] demonstrated that circulating immune cells from ACLF patients with poor clinical outcomes exhibit severe mitochondrial dysfunction and compromised cellular energetic. Concurrently, the coordinated collapse of App and Cd55 pathway protective mechanisms in our model suggests that cellular metabolic collapse and homeostatic failure are synchronized events across both parenchymal and non‐parenchymal networks during ACLF progression.

B cells play an important but long‐overlooked role in ACLF pathogenesis. The recent application of single‐cell transcriptomics has greatly advanced our understanding of B cell heterogeneity and functional remodeling in ACLF. Wang et al. [43] performed single‐cell sequencing on peripheral blood from HBV‐ACLF patients, identified six B cell subsets, and found that naïve B cells were significantly expanded and enriched for inflammatory response pathways, suggesting a pro‐inflammatory function in ACLF. Another study characterizing intrahepatic B cells in ACLF showed that, compared with cirrhosis and healthy controls, the proportion of naïve B cells was reduced in ACLF livers, whereas activated memory B cells, atypical memory B cells, IgM^+^ memory B cells, and plasma cells were increased; these B cell subsets exhibited enhanced activation and functional alterations, with intrahepatic plasma cells highly expressing PD‐L2 and secreting more granzyme B and IL‐10 [44]. Notably, B cell subset abnormalities are not limited to the liver; Cardoso et al. [45] found that in patients with acutely decompensated cirrhosis, double‐negative B cell frequencies were elevated in ACLF patients, whereas a reduction in transitional B cells was significantly associated with 1‐year mortality, suggesting that B cell subset composition may have prognostic value. In this study, we performed high‐resolution reclustering of the hepatic B‐cell compartment in ACLF mice and identified four transcriptionally distinct subsets with significant redistribution compared to controls. Pseudotime trajectory analysis revealed an ordered developmental progression, suggesting that ACLF drives B‐cell lineage differentiation toward specific effector states. The expansion of B cells and enrichment of Lgals9–Ighm signaling between ECs and B cells in our interactome analysis raise an intriguing hypothesis: endothelial‐derived Lgals9 may directly shape B cell recruitment and functional polarization in the ACLF liver. Galectin‐9, as a multifunctional β‐galactoside‐binding lectin, plays an important role in regulating liver inflammation; it has been shown that Gal‐9 ameliorates hepatocyte injury and local inflammatory infiltration in a mouse model of ConA‐induced fulminant hepatitis [46]. Further studies are required to dissect the precise role of the Lgals9–Ighm axis in ACLF—whether it promotes pro‐inflammatory B cell responses or serves as a compensatory immunoregulatory mechanism attempting to curb excessive inflammation.

To bridge the gap between bench and bedside, the potential microenvironmental discrepancies between our experimental murine model and real‐world human ACLF driven by diverse etiologies (such as HBV or MASLD) must be carefully evaluated. Our animal model, established via sequential chemical injury and acute decompensation, primarily mimics a state of sterile, toxic‐induced fibrotic background with superimposed acute inflammatory flares, representing a common convergent pathway of hepatic architectural and vascular collapse [47]. In the clinical setting, however, the intrahepatic immune and metabolic ecosystems are profoundly dictated by specific underlying etiologies. For instance, in HBV‐ACLF, the hepatic niche undergoes dramatic viral‐driven mitochondrial dysfunction that severely impairs fatty acid β‐oxidation and oxidative phosphorylation, driving a unique immune and metabolic remodeling [48]. This viral‐driven phenotype is further orchestrated by the dynamic polarization of hepatic macrophages, where resident Kupffer cells are rapidly depleted and replaced by virus‐primed monocyte‐derived macrophages that accelerate severe tissue destruction [49]. Conversely, in clinical ACLF cohorts driven by non‐viral etiologies such as MASLD, the hepatic microenvironment is characterized more by lipotoxicity, chronic metabolic endotoxemia, systemic immune exhaustion, and a widespread metabolomic crisis linked to inflammation‐associated mitochondrial failure across multiple organs [50, 51]. While our murine model successfully captures the core common denominators of ACLF—namely, severe endothelial dysfunction, localized mitochondrial stress, and the loss of homeostatic signals like CD55 and APP—the precise kinetics of the Lgals9–Ighm axis and B‐cell polarization may reflect the baseline immune‐metabolic imprinting unique to each specific etiology, underscoring the need for etiology‐specific validation in future clinical translational studies.

Several limitations of this study should be acknowledged. First, the mouse ACLF model does not fully recapitulate the etiological heterogeneity of human disease, and key findings (e.g., the Lgals9–Ighm axis) require validation in human tissues. Second, although our computational inference coupled with spatial co‐localization offers compelling evidence of physical proximity and potential interaction, a main limitation is the lack of direct genetic or pharmacological functional validation for the predicted Lgals9–Ighm axis; future in vitro co‐culture assays and in vivo targeting studies are warranted to fully elucidate its downstream signaling cascades. Third, single‐cell data represent static snapshots; longitudinal sampling would help reveal the temporal evolution of cellular communication. Fourth, the functional consequences of Cd55 and APP pathway suppression need to be elucidated through interventional studies.

Despite these limitations, our study provides valuable information. We have mapped a single‐cell atlas of NPCs in ACLF livers. We identified ECs as important drivers of cell–cell communication and pinpointed the Lgals9–Ighm axis as a potential link between endothelial dysfunction and immune cells, such as B cells. Moreover, the pathogenesis of ACLF involves not only the gain of pro‐inflammatory signals but also active loss of homeostatic protective mechanisms such as CD55 and APP. These findings suggest that the cellular communication network may represent a potential therapeutic target for this syndrome.

## Author Contributions

Tangwei Mou and Shaoyou Li conceptualized and designed the study, and provided overall study supervision. Jie Jia conceptualized and performed the bioinformatics and ligand–receptor interaction analysis. Yuehong Dong and Yu Zhao conducted the animal model construction, tissue processing. Tangwei Mou and Jie Jia executed the multiplex immunofluorescence methodology and quantitative image analysis. Tangwei Mou and Jie Jia contributed to the acquisition, interpretation of data, and drafting of the manuscript. Shaoyou Li and Tangwei Mou participated in the critical revision of the manuscript for important intellectual content.

## Funding

This work was supported by the Joint Special Fund of the Department of Science and Technology of Yunnan Province–Kunming Medical University (Grant 202301AY070001‐141), the Yunnan Fundamental Research Project (Grant 202301AT070132), the 535 Talent Project of the First Affiliated Hospital of Kunming Medical University (Grant 2023535Q09), the Yunnan Talent Support Plant (Grant 202505AS350006), Yunnan Key Laboratory of Organ Transplantation (Grant 202449CE340016), and the National Natural Science Foundation (Grant 31960161).

## Disclosure

The authors have nothing to report.

## Ethics Statement

Animal experimental procedures were performed in strict accordance with the ethical guidelines for animal welfare. The study protocol was officially approved by the Experimental Animal Management Committee of Kunming Medical University (Approval No. kmmu20221013). All animals received professional veterinary care throughout the study, which was conducted within certified facilities (No. SYXK [Dian]‐K2020‐0006) adhering to standardized biosecurity and institutional safety protocols.

## Conflicts of Interest

The authors declare no conflicts of interest.

## Supporting Information

Additional supporting information can be found online in the Supporting Information section.

## Supporting information


**Supporting Information 1** Figure S1: Full panel of liver histological images for all individual animals in control and ACLF groups. Figure S2: Complete histological, fibrotic, and apoptotic panels of liver tissues from all biological replicates. Figure S3: Statistical evaluation of individual cell population proportions between control and ACLF groups. Figure S4: Expression pattern of Lgals9 across endothelial cell subtypes. Figure S5: GSVA pathway enrichment analysis of hepatic B‐cell subpopulations in Control and ACLF livers. Figure S6: Quantitative evaluation of DAPI‐based total cell numbers and nuclear area percentages across two immunofluorescence panels. Figure S7: Quantitative comparison of additional positive cell populations between control and ACLF groups. Figure S8: Quantitative comparison of additional positive cell numbers between control and ACLF groups.

**Supporting Information 2** Table S1. B cell subcluster_specific gene.

## Data Availability

The raw scRNA‐seq data in this study have been deposited in the ScienceDB and accessible via the DOI: 10.57760/sciencedb.35640 and data access link https://www.scidb.cn/en/s/ErUFv2. The data that support the findings of this study are available in the Supporting Information of this article.
