# Supplementary material for: Single‐Cell Analysis of Hepatic Non‐Parenchymal Cells Reveals Endothelial Dysfunction and Altered Endothelial and B Cell Communication in Acute‐on‐Chronic Liver Failure
Source: J Immunol Res. 2026 Aug 31;2026:5421823. doi: 10.1155/jimr/5421823 (PMC13528445; doi:10.1155/jimr/5421823)
Supplement: Supplementary file 1 [file JIMR-2026-5421823-s001.pdf]

Supplementary materials

Single-cell analysis of hepatic non-parenchymal cells reveals endothelial dysfunction and altered endothelial and B cell communication in acute-on-chronic liver failure

Jia et al. Submission, 2026

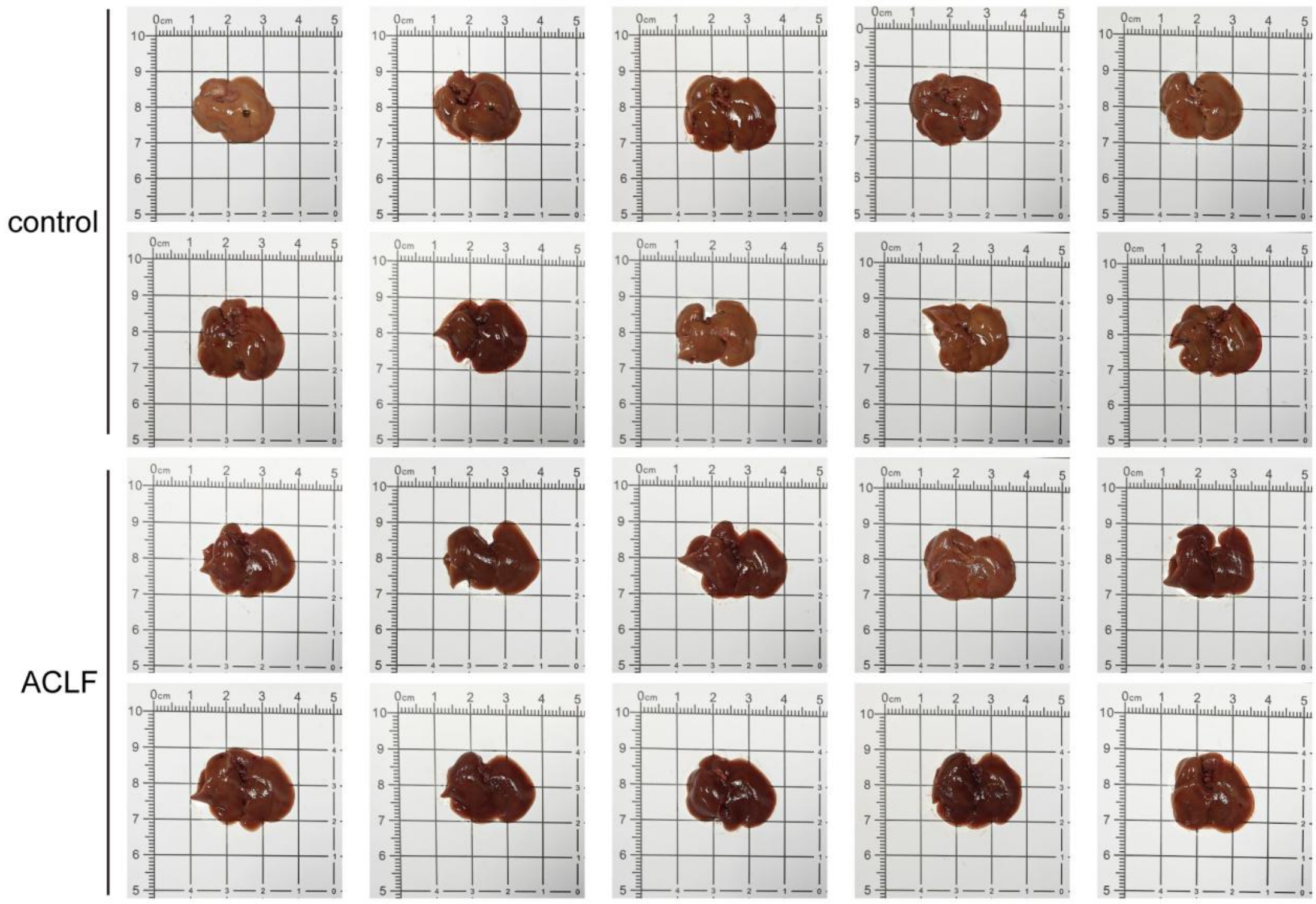

Figure S1. Full panel of liver histological images for all individual animals in control and ACLF groups. ACLF: acute-on-chronic liver failure. (n = 10). (related to Figure 1C).

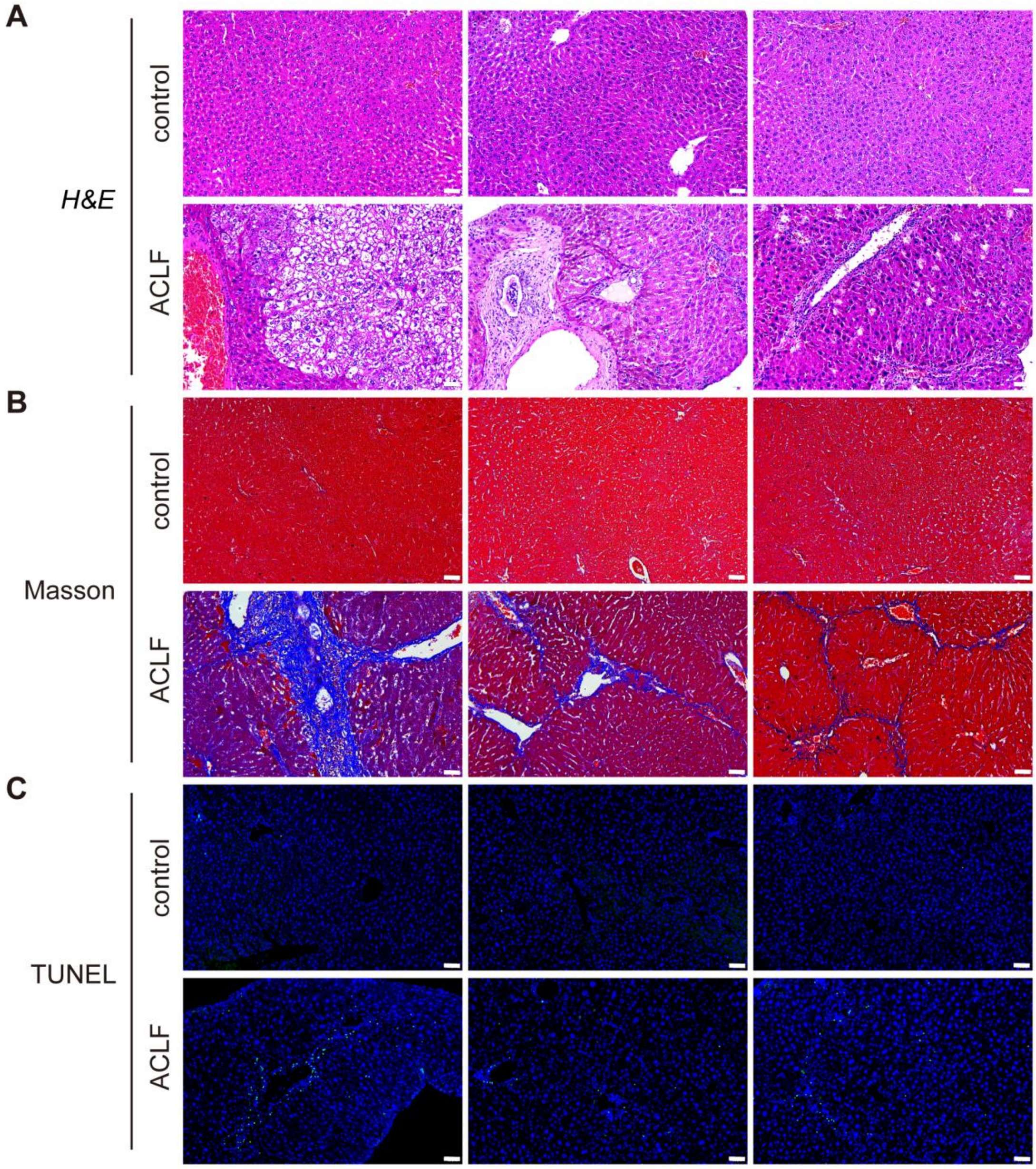

Figure S2. Complete histological, fibrotic, and apoptotic panels of liver tissues from all biological replicates. A. *H&E* staining: Complete sections of hematoxylin and eosin staining showing the overall hepatic lobular architecture, inflammatory cell infiltration, and necrosis across all biological replicates. (n = 3). **B.** Masson's trichrome staining: Sections showing collagen deposition (blue) and hepatic fibrotic background across all biological replicates. (n = 3). **C.** TUNEL staining: Sections showing cell apoptosis (green) within the liver tissue across all biological replicates. (n = 3). Scale bars = 50 μm. (related to Figure 1C).

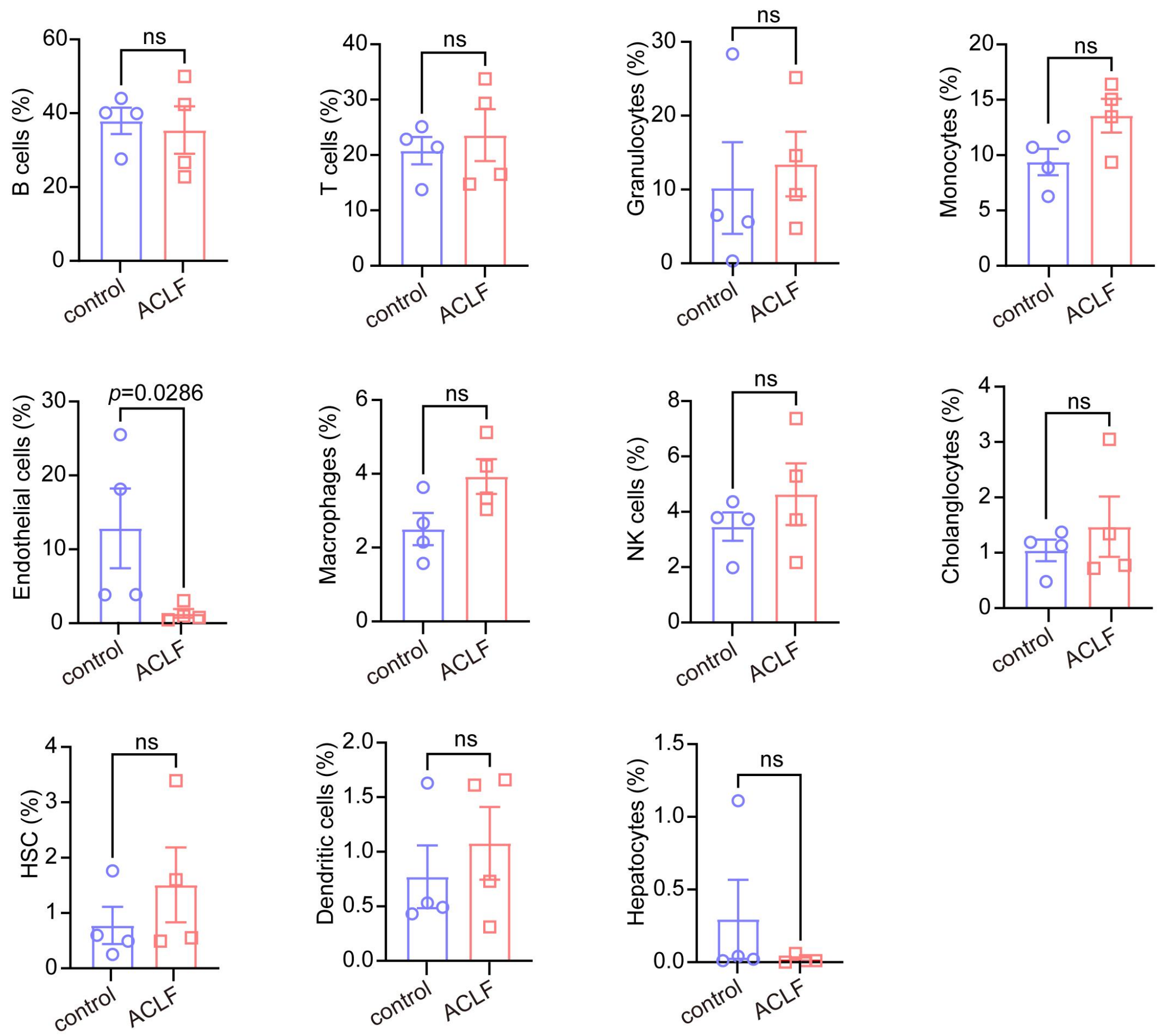

Figure S3. Statistical evaluation of individual cell population proportions between control and ACLF groups. A. Bar graphs showing the cell populations between control (blue) and ACLF (red) groups. Each dot represents an individual biological replicate (n = 4). Data are expressed as mean ± SEM. Statistical significance was calculated using unpaired Student's *t-test*, with the exact *p*-values indicated directly above each comparison. ns = not significant. (related to Figure 2F).

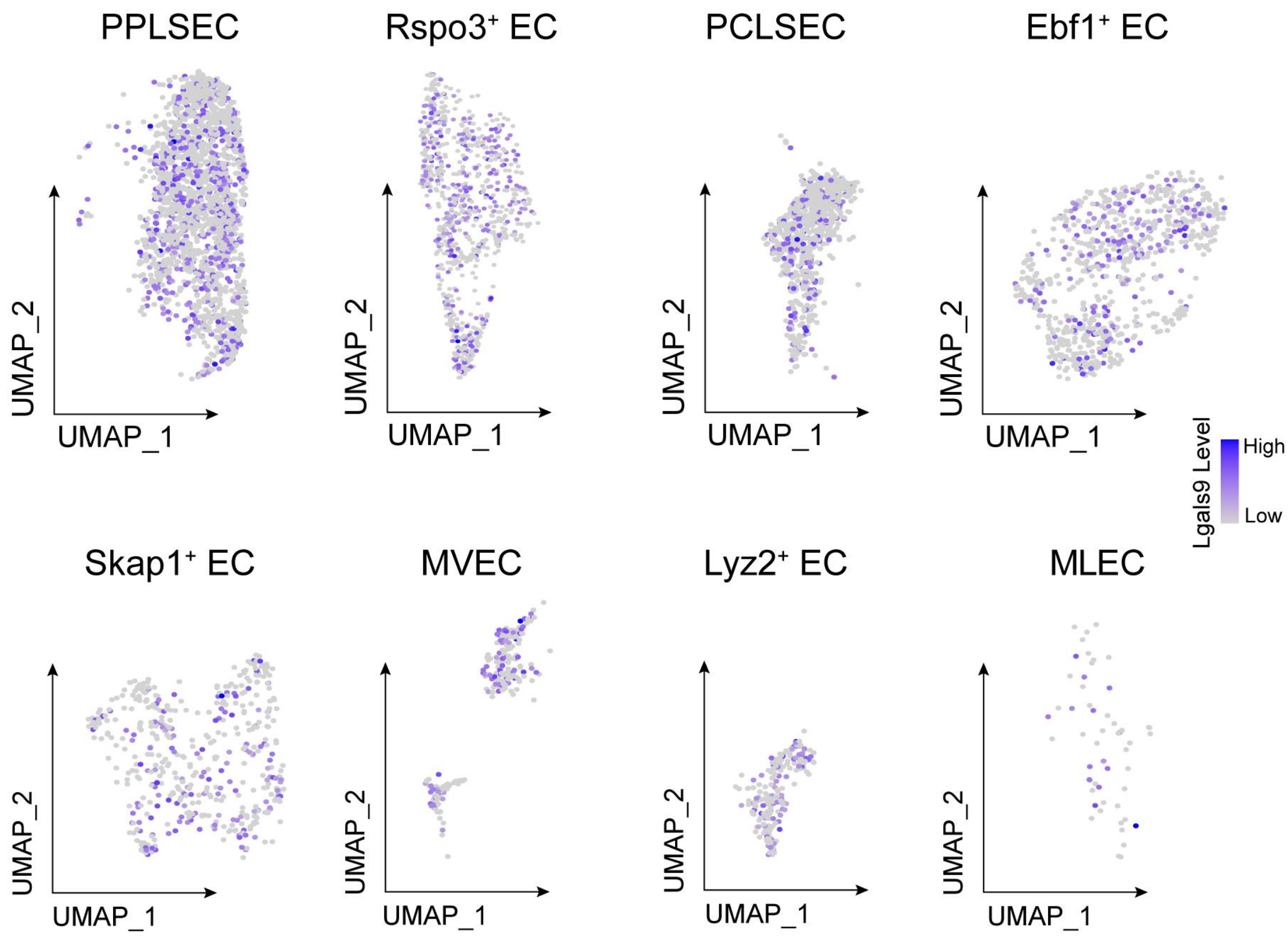

Figure S4. Expression pattern of Lgals9 across endothelial cell subtypes. A. UMAP feature plots showing the normalized gene expression levels of Lgals9 across eight distinct endothelial cell subpopulations. Color gradient represents the expression intensity of Lgals9 in single cells. (related to Figure 4).

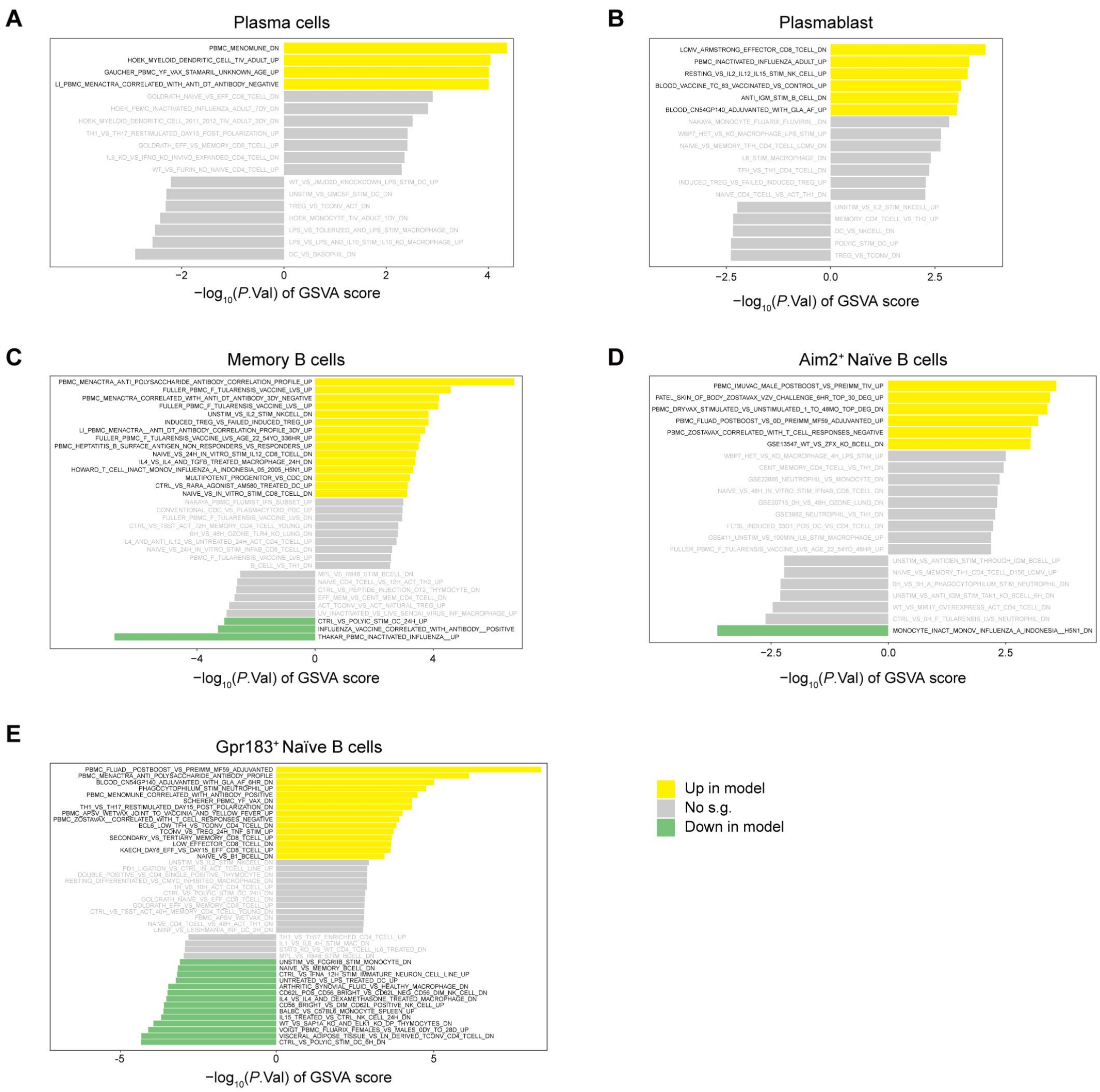

Figure S5. GSVA pathway enrichment analysis of hepatic B-cell subpopulations in Control and ACLF livers. A-E. Bar plot of representative GSVA terms enriched in in the B sub-clsuster control and ACLF Plasma B, Plasmablast, Memory B, Aim2^{+} Naïve B and Gpr183^{+} Naïve B cells)

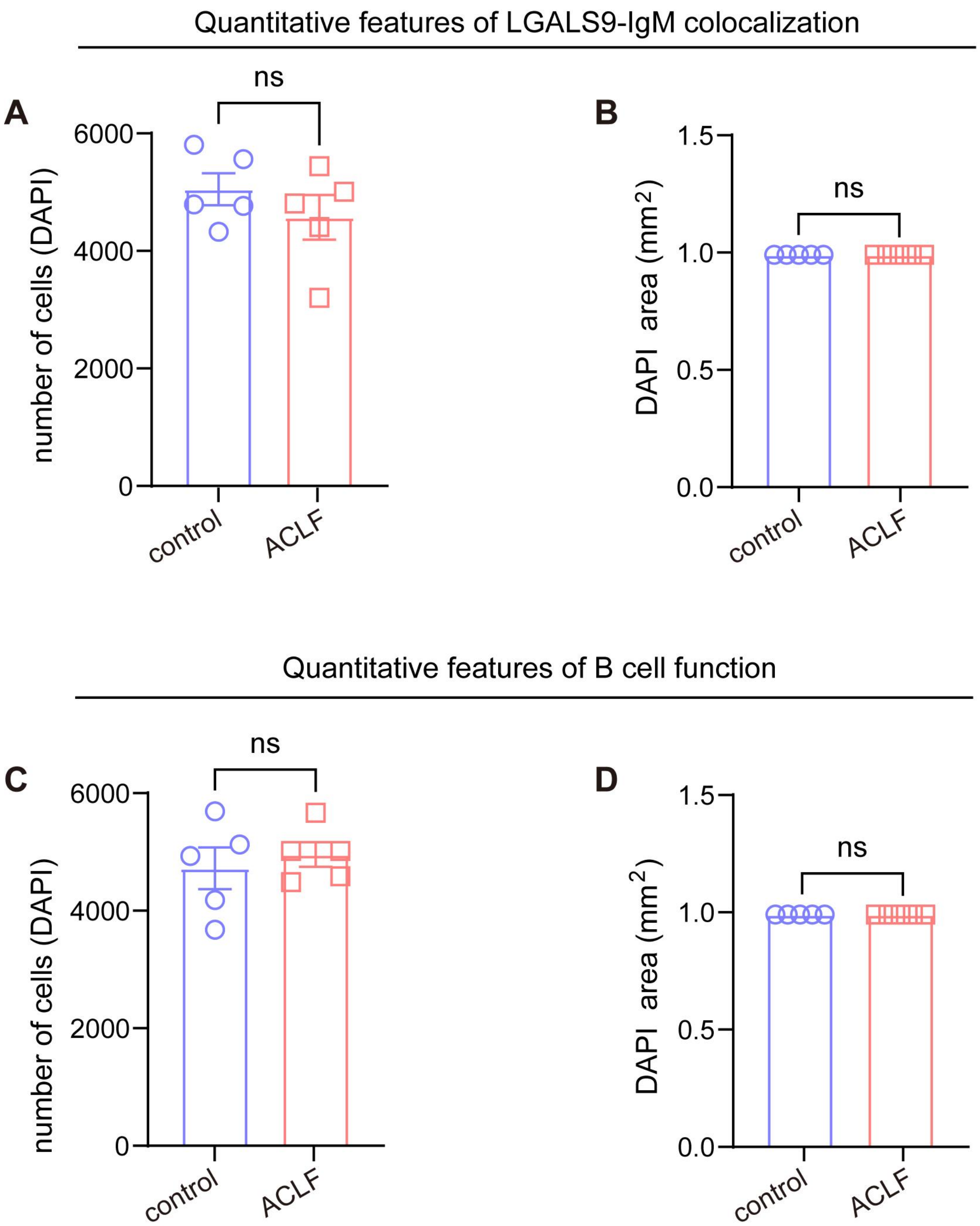

Figure S6. Quantitative evaluation of DAPI-based total cell numbers and nuclear area percentages across two immunofluorescence panels. A, B. Quantification in Panel 1: Bar graphs showing (**A**) total DAPI$^+$ nuclei and (**B**) DAPI$^+$ nuclear area. **C, D.** Quantification in Panel 2: Bar graphs showing (**C**) total DAPI$^+$ nuclei and (**D**) DAPI$^+$ nuclear area. Each dot represents an individual animal (n = 5), calculated as the mean value of 3 random fields per section. Data are shown as mean ± SEM. Unpaired Student's *t-test* was used. ns = not significant. (related to Figure 6C).

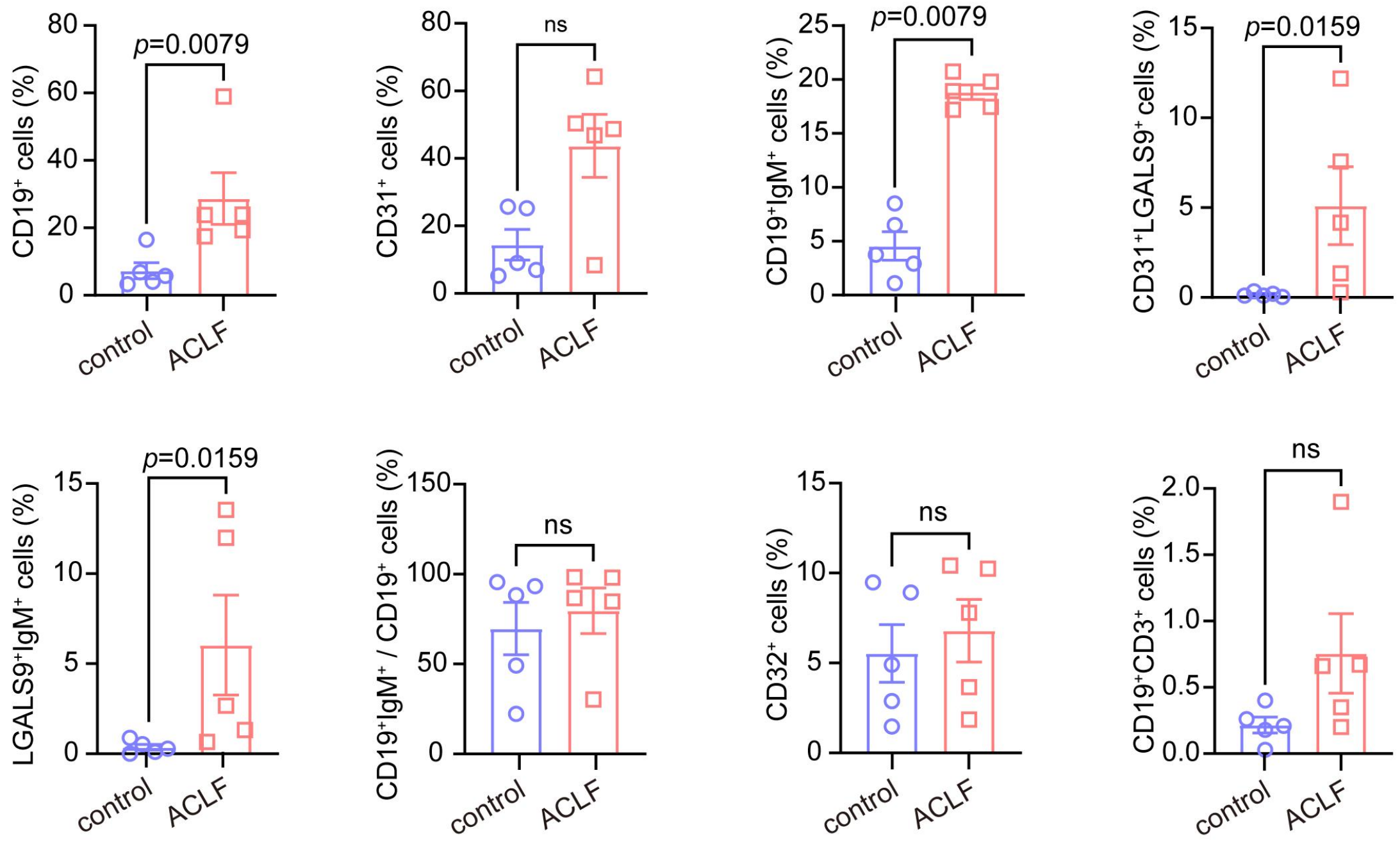

Figure S7. Quantitative comparison of additional positive cell populations between control and ACLF groups. A. Bar graphs showing the percentages of eight cell populations between control (blue) and ACLF (red) groups. Each dot represents an individual animal (n = 5), calculated as the mean value of 3 random fields per section. Data are shown as mean ± SEM. Unpaired Student's *t-test* was used, with exact p-values indicated above each column (or ns = not significant). (related to Figure 6C).

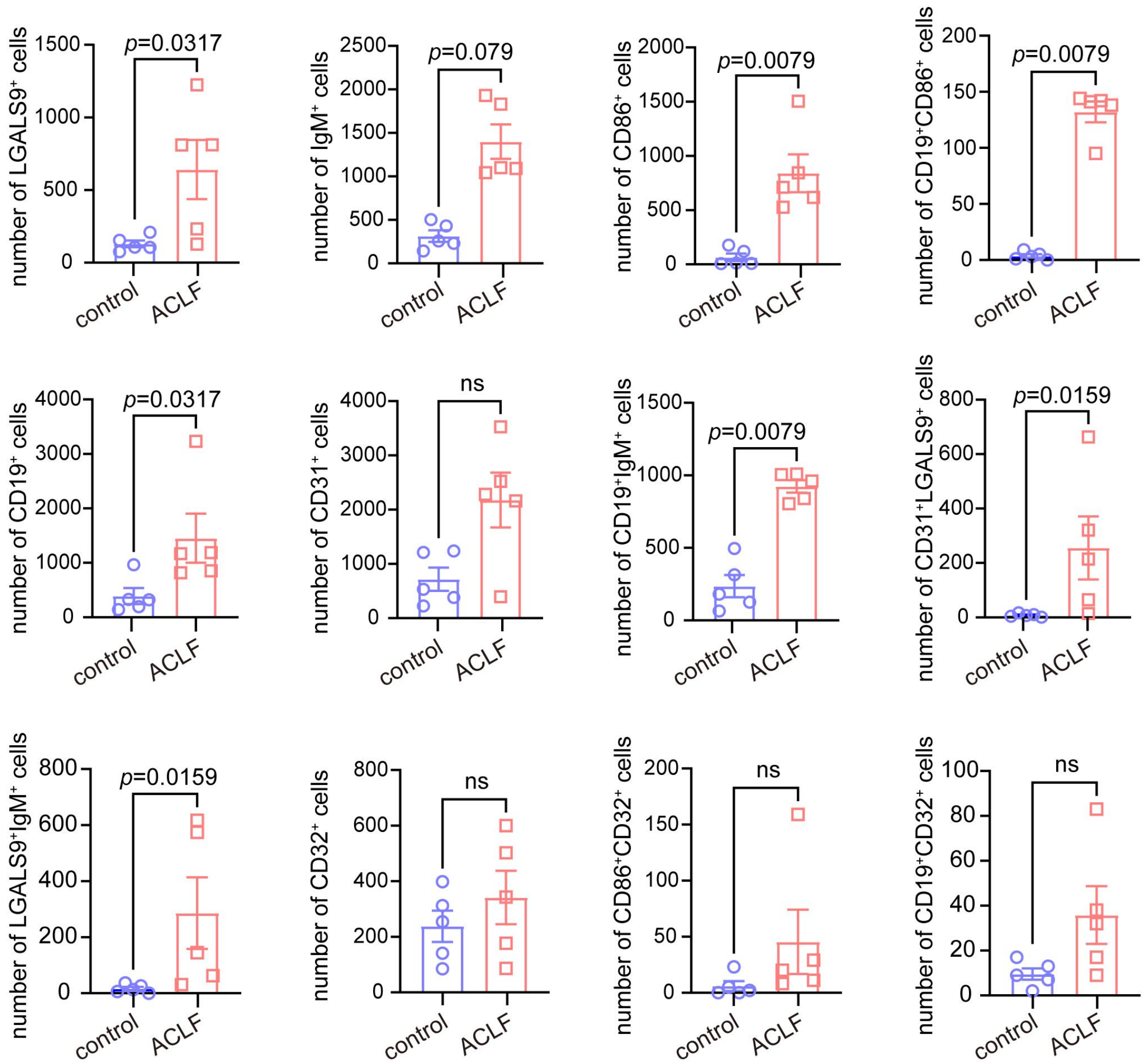

Figure S8. Quantitative comparison of additional positive cell numbers between control and ACLF groups. A. Bar graphs showing the number of 12 cell populations between control (blue) and ACLF (red) groups. Each dot represents an individual animal (n = 5), calculated as the mean value of 3 random fields per section. Data are shown as mean ± SEM. Unpaired Student's *t-test* was used, with exact *p*-values (or ns = not significant) indicated above each column. (related to Figure 6C).
